# Water and beverage consumption among children age 4-13y in the United States: analyses of 2005–2010 NHANES data

**DOI:** 10.1186/1475-2891-12-85

**Published:** 2013-06-19

**Authors:** Adam Drewnowski, Colin D Rehm, Florence Constant

**Affiliations:** 1 Université Pierre et Marie Curie - Paris VI, Groupe Hospitalier Pitié-Salpêtrière, 47 boulevard de l’Hopital, 75013 Paris, France; 2 Center for Public Health Nutrition, University of Washington, Box 353410, 98195 Seattle, WA, USA; 3 Nestlé Waters France, 12 boulevard Garibaldi, 92130 Issy-les-Moulineaux, France

**Keywords:** Drinking water, Water consumption, Children, Adequate hydration, Beverages, Dietary surveillance

## Abstract

**Background:**

Few studies have examined water consumption patterns among US children. Additionally, recent data on total water consumption as it relates to the Dietary Reference Intakes (DRI) are lacking. This study evaluated the consumption of plain water (tap and bottled) and other beverages among US children by age group, gender, income-to-poverty ratio, and race/ethnicity. Comparisons were made to DRI values for water consumption from all sources.

**Methods:**

Data from two non-consecutive 24-hour recalls from 3 cycles of NHANES (2005–2006, 2007–2008 and 2009–2010) were used to assess water and beverage consumption among 4,766 children age 4-13y. Beverages were classified into 9 groups: water (tap and bottled), plain and flavored milk, 100% fruit juice, soda/soft drinks (regular and diet), fruit drinks, sports drinks, coffee, tea, and energy drinks. Total water intakes from plain water, beverages, and food were compared to DRIs for the US. Total water volume per 1,000 kcal was also examined.

**Results:**

Water and other beverages contributed 70-75% of dietary water, with 25-30% provided by moisture in foods, depending on age. Plain water, tap and bottled, contributed 25-30% of total dietary water. In general, tap water represented 60% of drinking water volume whereas bottled water represented 40%. Non-Hispanic white children consumed the most tap water, whereas Mexican-American children consumed the most bottled water. Plain water consumption (bottled and tap) tended to be associated with higher incomes. No group of US children came close to satisfying the DRIs for water. At least 75% of children 4-8y, 87% of girls 9-13y, and 85% of boys 9-13y did not meet DRIs for total water intake. Water volume per 1,000 kcal, another criterion of adequate hydration, was 0.85-0.95 L/1,000 kcal, short of the desirable levels of 1.0-1.5 L/1,000 kcal.

**Conclusions:**

Water intakes at below-recommended levels may be a cause for concern. Data on water and beverage intake for the population and by socio-demographic group provides useful information to target interventions for increasing water intake among children.

## Background

Drinking plain water is an effective way to ensure adequate hydration [1,2]. Drinking plain water instead of caloric beverages may also help reduce dietary energy density and help in the management of body weight [3-6]. Water from beverages and foods - more than any macronutrient - is the key determinant of the energy density of the diet [7].

Hydration needs of children are a matter of public health concern [8,9]. Adequate intakes (AI) for water are defined on the basis of three factors: observed water intakes in population groups, desirable water volumes per 1,000 kcal, and desirable osmolality values in urine [8,10,11]. The US Institute of Medicine (IOM) AI recommendations for water are 1,700 mL/d for boys and girls in the 4-8y age group and 2,100 mL/d for girls and 2,400 mL/d for boys in the 9-13y age group [12].

The desirablewater-to-energy ratio is thought to be ≥1.0 L per 1,000 kcal [12]. In the US, the IOM Recommended Daily Allowances (RDA) Subcommittee set the standard water reference value for adults at 1.0 L per 1,000 kcal of energy expenditure [12]. That requirement could be increased to 1.5 L/1,000 kcal, depending on activity level and water loss. Guidelines issued by the European Food Safety Authority (EFSA) [10] specify that the total available water intakes should be 1.5 L/1,000 kcal for infants, 1.2 L/1,000 kcal for toddlers, and 1.0 L/1,000 kcal for adults. EFSA did not provide reference values for water intakes per 1,000 kcal for children [10].

The established Dietary Reference Intake (DRI) values for water are based on water obtained from plain drinking water (tap and bottled), water from other caloric and non-caloric beverages, and moisture from foods [12]. The DRIs were established by the IOM mostly to prevent the adverse effects of dehydration. Despite the focus on hydration and de-hydration in many official reports [10,12], some studies have shown that plain water consumption is associated with better diets, better health behaviors, and lower burden for chronic disease [4,13]. Park et al. [14] used data from the 2010 National Youth Physical Activity and Nutrition Study for 11,049 students in grades 9–12 to explore links between plain water consumption, socio-demographic characteristics, dietary habits, and selected health behaviors. In those adolescents, low water intake was associated with poor diet quality and physical inactivity.

With some exceptions [14-16], very few studies have explored the consumption of plain water in representative samples of US children. The present analyses were conducted using nationally representative National Health and Nutrition Examination Survey (NHANES) data for children 4-13y from 2005–2010. Estimates of total dietary water from all sources, including plain water, from other beverages such as juices and milk, and from moisture in foods were compared to the IOM recommendations for each age group. Energy intakes from beverage and food sources were also examined. Lastly, the water per calorie ratio (L/1000 kcal) was compared to desirable values by gender and age group.

## Methods

### Dietary intake databases

The present analyses used data from three cycles of the nationally representative NHANES, corresponding to 2005–2006, 2007–2008 and 2009–2010. The National Center for Health Statistics has obtained IRB approval for all cycles of NHANES and the data has been made available for public use [17]. The three NHANES cycles provided us with a nationally representative sample of 4,766 children age 4-13y.

These NHANES cycles were selected for two reasons. First, the collection of data on tap and bottled water consumed as a beverage only began in 2005 as part of the 24-hour recall [18]. In previous NHANES cycles, information about water was not collected in a comparable manner. Second, the 2005–2010 NHANES cycles included two 24-hour recalls for most respondents, making the data more representative of the usual dietary pattern of participants. The analyses were limited to respondents who completed two valid 24-h recalls. For analyses, the two-day mean was used.

The NHANES 2005–2010 surveys were based on 2 nonconsecutive 24-hour dietary recalls, with respondents listing the types and amounts of all food and beverages consumed in the preceding 24 hours. The first dietary recall was completed at a mobile examination center with a trained dietary interviewer, while the second recall was completed over the telephone some days later. For children 6-11y, the child was the primary respondent, but a proxy respondent (i.e., parent or guardian) was present and able to assist. For children 12-13y, the child was the primary source of dietary recall information, but could be assisted by an adult who had knowledge of the child’s diet [18-20].

The two-day mean should yield a reasonable estimate of mean intakes for populations or large sub-populations, but results in mis-estimating the proportion above or below a threshold value, such as the percent of children consuming too little water based on the DRI values. Therefore the presented proportions of children failing to meet the water AI should be interpreted as the lower-bound of the estimated proportion.

### Plain water and beverage consumption and energy intakes from beverages and foods

Beverages were classified into nine broad groups, as follows: water (bottled or tap), milk (including flavored), fruit juice (100%), soda/soft drinks (regular and diet), fruit drinks, sports drinks, coffee and coffee beverages, tea, and energy drinks.

The NHANES 24-hour recalls for each respondent provided information on the amount in grams of each food and beverage consumed. All results presented are for mL of water content from selected beverages, not intakes by volume (e.g., we present mL of water in milk, not mL of milk consumed), as information on beverage volume is not provided in the NHANES data. Energy intakes from beverages and foods were evaluated in a similar manner, based on energy values from the Food and Nutrition Database for Dietary Studies corresponding to each cycle of NHANES. The same categories of beverages were used for analyses of energy, but included non-beverages as an additional source of energy.

### Statistical Analyses

Analyses evaluated the survey-weighted mean 2-day intake of total water for the entire child population and by age group, gender, race/ethnicity and family income-to-poverty ratio. The age groups were 4-8y and 9-13y and race/ethnicity was defined by self-report as non-Hispanic white, non-Hispanic black, Mexican-American, other Hispanic and mixed race/other. Four categories of family income-to-poverty ratio (defined as the ratio of family income to the Federal Poverty Level [FPL] and adjusted for the number of adults and children in the household) were created. In 2010, the FPL for a family of four was $22,050 in the contiguous United States. These categories were defined as <1.0 (family income less than 100% of the FPL), 1.0-1.99, 2.0-3.49, and ≥3.5 (family income greater than or equal to 350% of the FPL). Analyses evaluated the consumption of tap and bottled water separately for the entire population and the sub-groups previously described. Mean values of total water and energy were estimated for beverage types previously described and the population proportion was estimated. Since the mean of two 24-hour recalls does not represent the habitual intakes of an individual, analyses of the percent of children failing to meet the DRIs represent an estimate of the lower-bound of the number of children who fail to meet the recommended intake levels. All analyses accounted for the complex NHANES stratified multistage sampling design and for the over-sampling of some groups (e.g., non-Hispanic black and Hispanic population). The analysis also accounts for survey non-response and all results are representative of the US child population from 2005–2010. Analyses were conducted using Stata 11.0 (College Station, TX).

## Results

### Plain water consumption

Data presented in Table 1 shows the consumption of plain water as a beverage (tap or bottled water) for the entire child population, and by age and socio-demographic group. The percentage of children reporting consuming plain water as a beverage on both NHANES recall days was 72%.

**Table 1:** Consumption of plain water (tap and bottled) in mL among children age 4-13y by socio-demographic group, NHANES 2005–2010

	**N**	**Plain water**	**Tap water (mL)**	**Bottled water (mL)**
All Children	4766	431.0 (13.1)	257.2 (12.4)	173.9 (9.4)
Age group				
4-8y	2327	364.9 (13.5)	226.8 (14.8)	138.1 (10.3)
9-13y	2440	496.1 (19.4)	287.0 (17.8)	209.0 (12.4)
P-difference		<0.001	0.009	<0.001
Gender				
Boys	2398	423.1 (15.8)	269.6 (15.1)	153.5 (9.7)
Girls	2369	439.0 (17.1)	244.6 (16.6)	194.4 (13.5)
P-difference		0.43	0.21	0.006
Race/ethnicity				
Mexican-American	1422	374.4 (15.6)	163.6 (13.9)	210.8 (12.2)
Other Hispanic*	408	417.0 (59.9)	185.9 (23.8)	231.1 (46.1)
Non-Hispanic White	1462	456.5 (23.0)	296.9 (20.8)	159.6 (13.6)
Non-Hispanic Black	1182	373.9 (16.8)	197.8 (15.5)	176.1 (13.4)
Other race – including mixed race*	293	461.4 (43.5)	295.5 (35.9)	165.9 (32.8)
P-difference^1^		0.012	<0.001	0.007
Family income-to-poverty ratio				
<1	1403	375.0 (19.6)	224.3 (19.0)	150.8 (12.2)
1-1.99	1257	426.9 (26.3)	244.2 (26.0)	182.7 (18.0)
2-3.49	871	425.2 (25.6)	258.6 (19.0)	166.6 (18.1)
≥3.5	965	470.2 (32.3)	290.8 (28.8)	179.4 (16.9)
P-difference		0.08	0.26	0.35

Values presented are survey-weighted means and standard errors (SE) in parentheses.

*Interpret cautiously due to small numbers.

^1^P-value of difference omitting the two smaller categories was 0.015 indicating that among the groups with sufficient numbers, there is a significant difference in water consumption.

On average, children 4-13y drank 431 mL/d of water as a beverage. Younger children (4-8y) drank 365 mL/d while older children (9-13y) drank 496 mL/d. Boys and girls did not differ in the amounts of water consumed.

There was a strong effect of race/ethnicity on total water consumption. Non-Hispanic white children consumed more plain water on average, as compared to Mexican-American and non-Hispanic black children. Socio-economic status was marginally associated with water intakes. Children living in higher-income households were more likely to consume water as a beverage than were children living in lower-income households (p = 0.08).

The percentage of children reporting consumption of tap water as a beverage on both NHANES recall days was 65% whereas the percentage of children reporting bottled water consumption was 43%. Overall, about 60% of water consumed as a beverage was tap water and 40% was bottled water.

Patterns of tap versus bottled water consumption were influenced by age, gender, and race/ethnicity. The absolute amounts of tap and bottled water are presented in Table 1. As a proportion of water consumed as a beverage, 58% and 62% of total water consumed as a beverage came from the tap among older and younger children respectively. Sixty-four percent of water consumed as a beverage for boys came from tap water compared to 56% for girls. Non-Hispanic white children consumed 65% of their water as a beverage from the tap, while 44% and 53% of water consumed as a beverage among Mexican-American and non-Hispanic black children respectively, was from tap sources. There were no clear patterns in preference for tap vs. bottled water by family income.

### Water intakes from plain water, beverages, and foods

**Figure 1:**
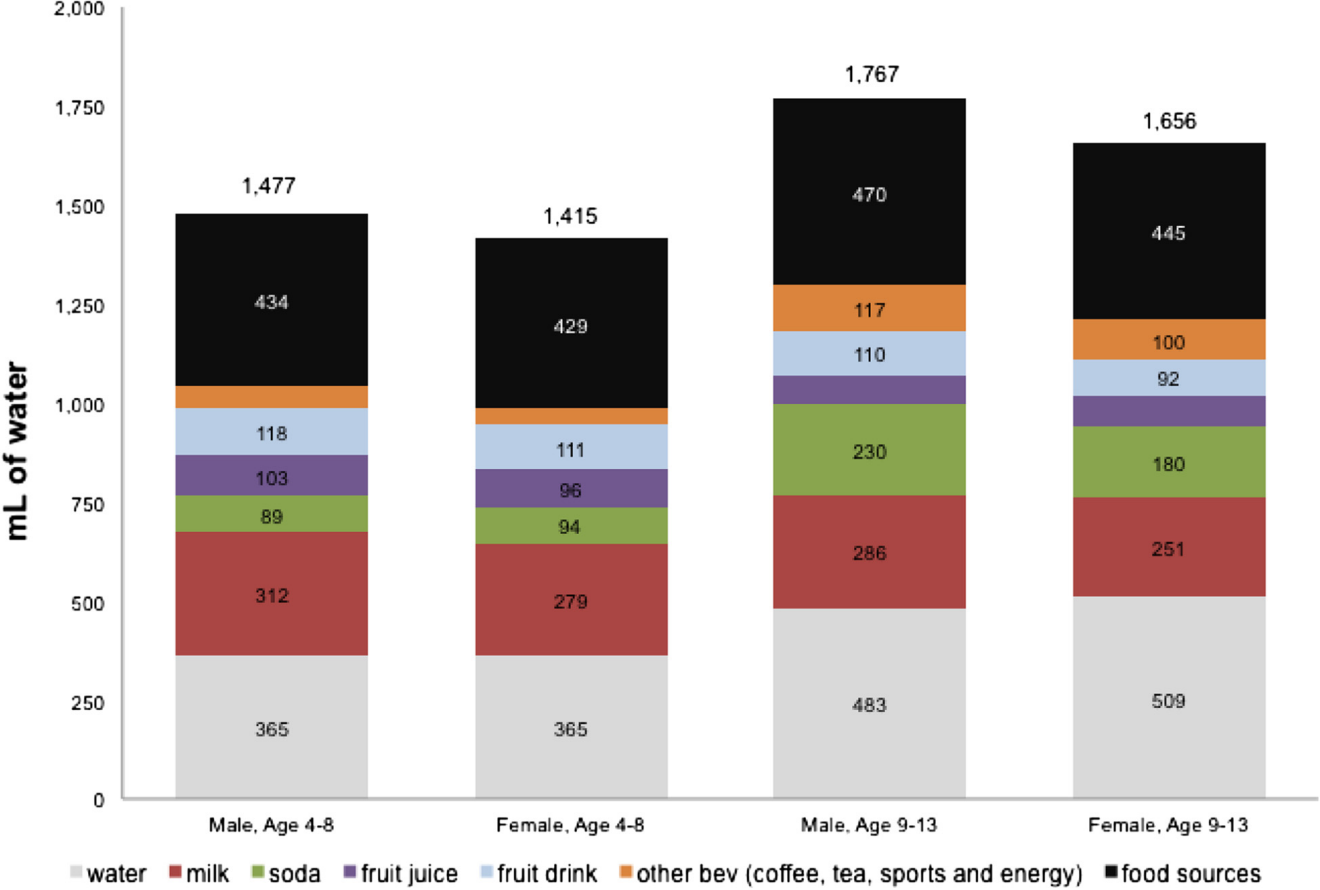
Daily water intakes from all sources by age group and gender, NHANES 2005–2010.

Figure 1 summarizes the principal sources of total dietary water by age group and gender. The chief sources were plain water, moisture from foods, milk, soda, fruit juices and fruit drinks and other beverages.

Table 2 shows the amounts of water from plain water, other beverages and foods by age group. Since milk was often used with cereal, results are presented for milk (total) and for milk consumed as a beverage (i.e. not with cereal).

**Table 2:** Volume of water (mL) from plain water, other beverages and from moisture in foods as consumed by children age 4-13y, overall and by age group, NHANES 2005–2010

	**Total 4-13y**		**Age 4-8y**		**Age 9-13y**	
	Mean (SE)^1^	% of total	Mean (SE)^1^	% of total	Mean (SE)^1^	% of total
Water	431.0 (13.1)	27.3	364.9 (13.5)	25.2	496.1 (19.4)	29.0
Milk and milk beverages	282.0 (5.9)	17.8	295.8 (5.9)	20.4	268.5 (10.4)	15.7
Excluding milk w/cereal	212.5 (5.8)	13.4	225.6 (6.0)	15.6	199.6 (9.9)	11.7
Fruit juice	87.2 (3.9)	5.5	99.3 (3.8)	6.9	75.2 (5.7)	4.4
Soda	148.6 (7.1)	9.4	91.4 (5.5)	6.3	204.8 (11.6)	12.0
Diet soda	19.7 (2.3)	1.2	12.2 (1.9)	0.8	27.0 (4.1)	1.6
Regular soda	128.9 (6.7)	8.2	79.2 (5)	5.5	177.8 (10.7)	10.4
Fruit drink	107.6 (4.8)	6.8	114.6 (5.5)	7.9	100.6 (6.2)	5.9
Low-calorie fruit drink	28.3 (3.5)	1.8	32.9 (4.6)	2.3	23.7 (3.3)	1.4
Regular fruit drink	79.3 (3.1)	5.0	81.8 (3.8)	5.7	76.9 (4.3)	4.5
Sports drink	37.2 (4.7)	2.4	25.6 (4.7)	1.8	48.5 (7.5)	2.8
Coffee	4.6 (0.9)	0.3	1.8 (0.5)	0.1	7.3 (1.7)	0.4
Tea	36.7 (4.7)	2.3	21.8 (2.7)	1.5	51.3 (8.1)	3.0
Energy drink	0.9 (0.4)	0.1	0.1 (0.1)	0.0	1.6 (0.9)	0.1
Water from beverages	1135.8 (16.8)	71.4	1015.4 (14.7)	70.2	1254 (29.1)	73.3
Water from food^2^	444.6 (6.0)	28.6	431.4 (7.2)	29.8	457.5 (8.2)	26.7
Total daily water	1580 (16.5)	100.0	1447 (14.7)	100.0	1711 (29.6)	100.0

^1^SE are standard errors.

^2^Milk consumed with food is included as a beverage.

For all children, plain water and other beverages contributed 71% of daily water intakes. Among beverages, plain water as a beverage provided the most daily water, followed by milk and soda. Moisture in foods contributed a similar amount of total water as water consumed as a beverage.

Sugar-sweetened beverages (as opposed to diet beverages) dominated the soda category among children. After moisture in foods, water as a beverage and milk, soda was the most important source of total water, followed by fruit drinks and fruit juices. Tea and sports drinks contributed modest amounts of total water. No other beverage source contributed more than 0.5% of total water. Analyses by gender confirmed that boys and girls had comparable consumption patterns. Drinking water (tap or bottled) accounted for 26.1% of all dietary water among boys and 28.5% among girls (data not shown).

Analyses of plain water intakes by age reveal some differences in sources of water between older and younger children. Water as a beverage accounted for 29% of total water among older children and 25% among younger children. In addition, older children received a higher proportion of their total water from soda, sports drinks and tea, while younger children received a greater proportion of their total water from milk, fruit juice and fruit drinks.

The amounts of water and beverages consumed varied by race/ethnicity (data not shown). Among non-Hispanic white children, water consumed as a beverage accounted for about 28% of total water, while milk and soda were the most important beverage sources of total water, accounting for 18.6% and 10.1% of total water respectively. In this sub-population, moisture in foods accounted for 27% of total water. Non-Hispanic black children obtained 25.9% of their total water from water as a beverage. Milk was the third most important source of total water (14.2%), but contributed much less water when compared to other race/ethnicity groups. Fruit drinks contributed 11.9% and moisture in foods provided 29.3% of total water among non-Hispanic black children. Among Mexican-American children, 24.2% of total water came from water consumed as a beverage. Milk contributed 19.4% of total water, while fruit juice and fruit drinks accounted for 7.1% and 6.9% of total water respectively. Soda accounted for 9.1% of total water intake among Mexican-American children and moisture in foods provided 29.5% of total daily water.

### Energy intakes from beverages and foods

Table 3 shows the contribution of beverages to energy intakes of children by beverage category and age group. For all children, milk was the leading source of calories from beverages. For younger children, fruit juices were the second leading source of energy among all beverages. Fruit juices were replaced by soda as the second leading source of calories from beverages among older children.

**Table 3:** Contribution of foods and beverages to total energy intakes (kcal) among children age 4-13y, overall and by age group, NHANES 2005-2010

	**Total 4-13y**		**Age 4-8y**		**Age 9-13y**	
	Mean (SE)^1^	% of total	Mean (SE)^1^	% of total	Mean (SE)^1^	% of total
Milk	180.6 (3.6)	9.7	192.7 (3.9)	11.0	168.6 (6.3)	8.6
Excluding milk w/cereal	140.6 (3.6)	7.6	151.7 (3.8)	8.6	129.8 (6.0)	6.6
Soda	56.1 (2.9)	3.0	34.4 (2.1)	2.0	77.4 (4.7)	3.9
Fruit juice	47.5 (2.1)	2.6	54.5 (2.2)	3.1	40.6 (3.1)	2.1
Fruit drink	45.9 (1.7)	2.5	48.7 (1.8)	2.8	43.2 (2.4)	2.2
Sports drink	9.6 (1.1)	0.5	6.6 (1.1)	0.4	12.5 (1.7)	0.6
Tea	6.0 (0.8)	0.3	4.2 (0.6)	0.2	7.7 (1.2)	0.4
Coffee	0.8 (0.2)	0.0	0.2 (0.1)	0.0	1.3 (0.3)	0.1
Energy drink	0.4 (0.3)	0.0	0.1 (0.1)	0.0	0.8 (0.5)	0.0
Water	0.1 (0.0)	0.0	0.0 (0.0)	0.0	0.1 (0.1)	0.0
Total beverages	346.9 (4.9)	18.7	341.6 (4.5)	19.5	352.1 (9.4)	18.0
Total foods^2^	1512 (11.9)	81.3	1414 (13.2)	80.5	1608 (16.7)	82.0
**Total energy (kcal)**	**1859 (15.0)**	**-**	**1756 (14.9)**	**-**	**1960 (22.4)**	**-**

^1^SE are standard errors.

^2^Milk consumed with food is included as a beverage.

### Water intakes compared to IOM recommendations

**Figure 2:**
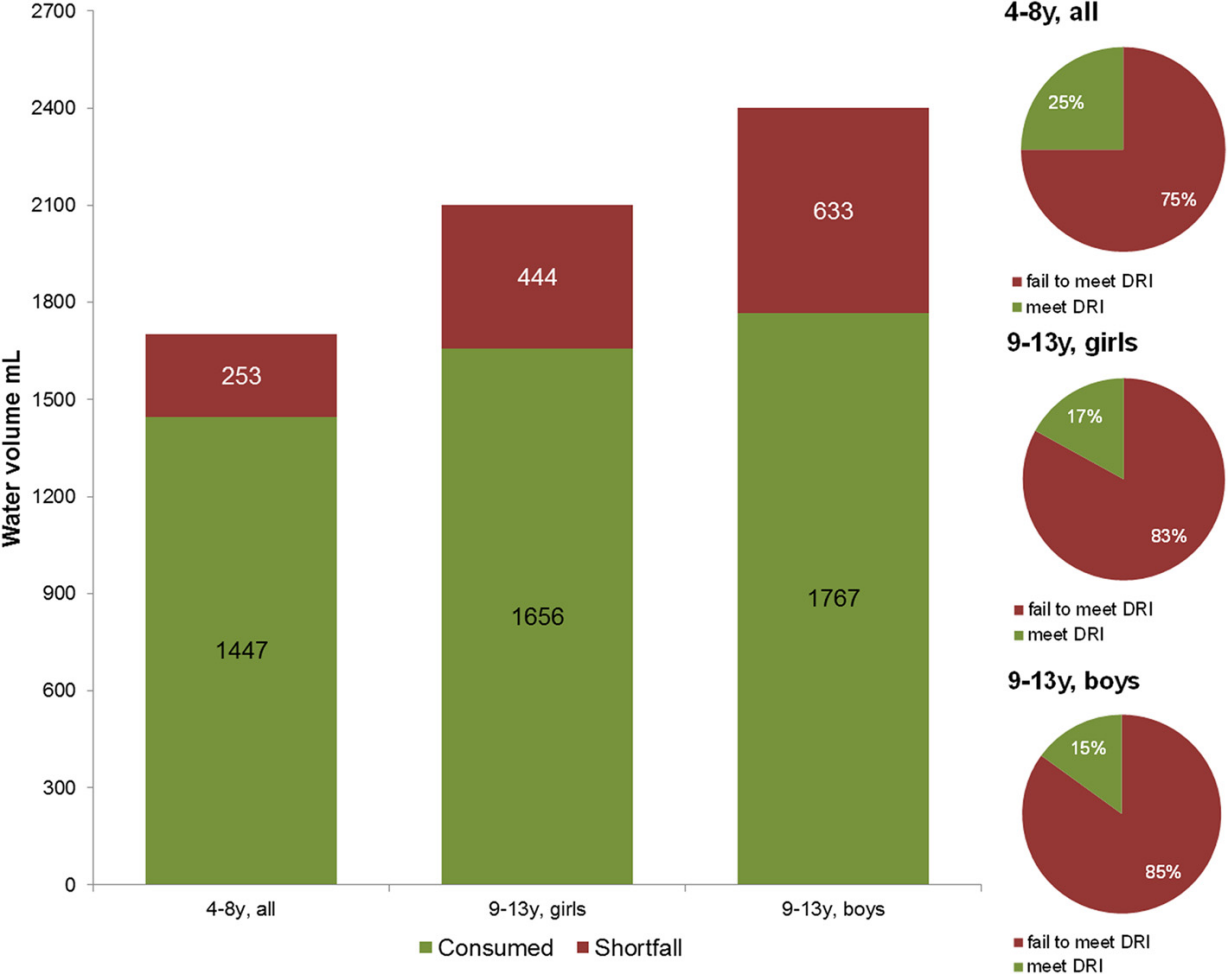
**Daily water intakes from all sources by age group and gender in relation to IOM recommendations (left panel).** The size of the shortfall for total water intake is indicated on the graph. The proportion of children by age group and gender who do or do not meet IOM recommendations is indicated in the right set of panels.

Total water intake, from drinking water, other beverages and moisture in foods, was then compared to age- and gender-specific DRI values published by the IOM. As shown in Figure 2, no group of children met the IOM recommendations. The shortfall in water consumption relative to the IOM AI values ranged from 253 mL/d to 633 mL/d. Depending on gender and age group, fewer than 15-25% of children met the IOM recommendations for total water intake.

**Figure 3:**
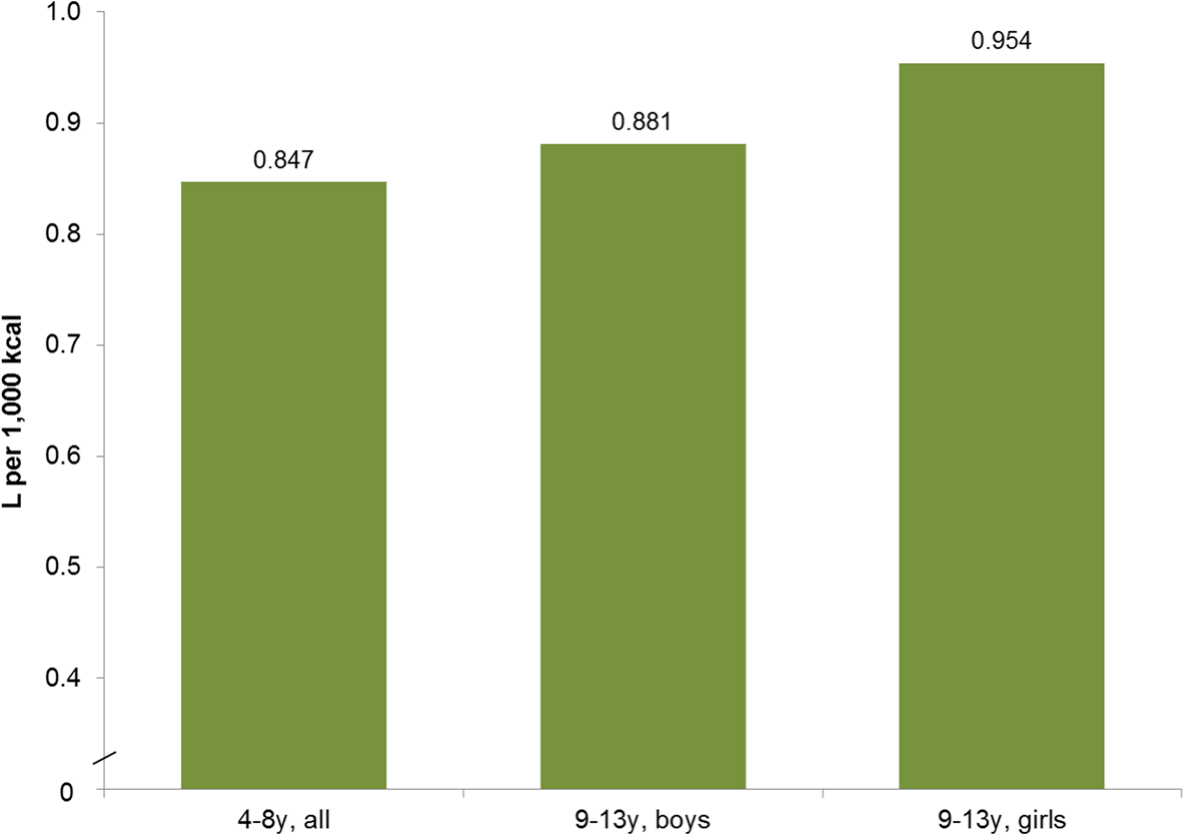
The mean of the ratio of water (L) per 1,000 calories by age and gender group, NHANES 2005–2010.

As indicated in Figure 3, the observed water volume per 1,000 kcal was between 0.85 and 0.95 L/1,000 kcal, short of the desirable values (≥1.0 L/1,000 kcal) recommended by the IOM [12] and EFSA [10].

## Discussion

These analyses of total water intakes from all sources, including tap and bottled water, were conducted among a representative sample of US children age 4-13y from 2005–2010 NHANES. The amounts of dietary water provided by plain water and by other beverages and foods were compared to AI values by gender and age group. The intent was to examine how close children and adolescents came to meeting the AI values, as defined by the IOM DRIs. According to the IOM, AI values may be used as goals for individual intakes [12], though there is much inter-individual variation. Water needs can be influenced by health status; physical activity or strenuous work; dietary factors, including sodium and protein intake; and environmental factors, such as temperature and humidity [2,10,12,21-23]. These additional factors need to be considered when evaluating adequate intakes at the individual level.

The majority of girls (83%) and boys (85%) age 9-13y failed to meet the IOM DRI value. Children aged 4-8y consumed about 15% less water on the average than the IOM DRIs and at least 75% failed to meet the IOM DRIs for water. Girls aged 9-13y were 444 mL/d short of meeting the IOM DRI recommendations for water, whereas boys aged 9-13y were 633 mL short. EFSA provides additional recommendations for total water intake, which are marginally different from the IOM DRI values (e.g., 1,600 mL/d for children 4-8y based on EFSA compared to 1,700 mL/d based on IOM DRIs) [10]. We conducted secondary analyses using the EFSA values. At least 72% of children age 4-13y failed to meet the EFSA guidelines. At least 69% of children 4-8y, 73% of girls 9-13y and 75% of boys 9-13y failed to meet the EFSA recommendations. The second criterion of adequate hydration, water volume per 1,000 kcal also fell short of desirable values. Whereas the standard IOM recommendation is at least 1.0 L per 1,000 kcal [12], the observed values were in the 0.85-0.95 L range, depending on age and gender.

Urine osmolality is another measure of adequate hydration, but it was obtained for only 1 cycle of NHANES data (2009–2010) and not evaluated in the present study [12]. A recent study of 548 children age 9-11y showed elevated urine osmolality (an index of hyperosmotic cell shrinkage) in more than 63% of schoolchildren in Los Angeles and New York [24]. Elevated urine osmolality was associated with not drinking water in the morning prior to going to school. Although 90% of the children had breakfast, 75% did not drink water at breakfast. A majority of participants (value not provided in the paper) reported consuming any food or a beverage other than water at the morning meal [24].

The present analyses of the observed water intakes relative to the indices of hydration suggest that children’s water consumption ought to be monitored more closely [25]. In 2010, EFSA published a 48 page report on water consumption alone [10], arguing that water is often disregarded in national and international recommendations or is very cursorily treated [10]. For example, the 2010 US Dietary Guidelines devoted only two pages to water, stating that most healthy people consumed adequate water to meet their needs [26]. Because water needs vary considerably by individual characteristics, the Dietary Guidelines Advisory Committee concluded that a minimum intake of water could not be set [27].

The present study provides valuable new data on children’s consumption of plain drinking water and other beverages. The data on water consumption by socio-demographic group may be useful in identifying population sub-groups that may benefit from targeted interventions to increase total water intake. The evaluation of tap versus bottled water by population sub-group provides additional data to support potential intervention strategies. Past studies have tended to focus on the contribution of beverages to energy and nutrient intakes, focusing variously on milk [28], fruit juices, and sweetened beverages [29,30]. In some cases, the consumption of caloric beverages was related to the children’s body weight [29]. Other studies [28] have made the point that some of the nutrient-poor beverages that the children were consuming could be replaced with more nutrient-dense options such as low-fat and fat-free milk.

Given the dearth of recent data on water and beverage consumption among US children, the present study fills a gap in the existing knowledge on water consumption patterns among US youth. As yet, there are no clear recommendations on the desirable water volumes per 1,000 kcal for children and adolescents. The scientific evidence cited in the 2010 Dietary Guidelines Advisory Committee Report referenced data from NHANES III showing that fluids provided 3.0 L/day for men and 2.3 L/day for women aged 19-30y [27]. Whereas fluids provided approximately 81% of total water intake, moisture in food provided the remaining 19%. Child-specific data were not provided.

Future guidelines on beverage consumption for children should take plain drinking water into account. This is particularly important given the size of the shortfall between observed intakes and DRI reference values. Total water intake can be increased in a number of ways. The most effective way would be to increase the consumption of plain water, either tap or bottled. In addition to promoting skim and low-fat milk consumption, the USDA now requires schools participating in the National School Lunch program to make free potable water available to students when meals are served [31-33]. In addition, as numerous recommendations to reduce intake of caloric beverages have emerged [26,34,35], it will be important to carefully monitor total water intake to determine if such policies and interventions may have a deleterious impact on total water intake.

In the present analyses of NHANES 2005–2010 data, non-beverage food sources accounted for 25-30% of total dietary water for most population groups, as opposed to 19% from previous reports [27]. Increasing consumption of low-energy-density foods with high water content (e.g., fruits/vegetables) is another way to increase water intakes. However, a modest increase in fruit and vegetable consumption is unlikely to have a major impact on adequate hydration; drinking water and beverages are more effective strategies.

It is important to mention that these data cannot be directly compared to those from cycles of NHANES prior to 2005, as the method for collecting water intake data has changed. Prior to 2005, water intake was measured after the dietary recall was completed. In more recent cycles (2005 onward), water was reported during the 24-hour recall in the same manner that any other food/beverage was reported [18-20]. Comparisons of water intake for the total adult population and adult population sub-groups from 1999–2004 and 2005–2006 reveal that estimated water intakes are generally 15% lower when comparing new (2005–2006) to old data (1999–2004) [36]. While this difference may be attributable to secular changes in water intake, they may also be explained by changes in data collection. It is unclear which approach is most valid for assessing water intake, but caution should be applied when comparing the results presented here to data collected prior to 2005, especially in interpreting whether children or any group consume less water now (2005–2010) than in the past. Given these issues, the work presented here focuses on comparisons between groups of children and to established reference values, as opposed to an evaluation of trends in water intake.

The present analyses had limitations. First, the NHANES data are based on self-report and are subject to random and systematic reporting errors. Proxy recall for younger children may be an additional source of error. Different diet recall methods used to collect the data (first day in-person and second day over the telephone many days later) may introduce mode effects into the estimate of water consumption. If water intakes were under-reported in the NHANES database, then the estimates presented here will over-estimate the percent of adults who fail to meet the recommended intakes. It is possible that many respondents under-reported water intakes due to drinking water events lacking saliency. This may be particularly problematic for events where little water was consumed (e.g., stopping at a drinking fountain) or when it was consumed casually without active choices being made by participant (e.g., repeatedly being refilled at a restaurant). As noted previously, since methods for data collection have changed the results presented here cannot be directly compared to those from previous cycles of NHANES. Despite these limitations, these data have a number of advantages as they represent a large, nationally representative data source that forms the basis for dietary surveillance in the US.

## Conclusions

The present analyses represent one of the few explorations of plain water consumption among US children and can be used to inform approaches to improve the overall quality of children’s diet and their hydration status. We observed that at least 75% of children failed to meet DRI reference values for water intake, which may warrant careful monitoring of total water intake in the coming years. Additional data presented here on water and beverage intake by socio-demographic group may be useful for focusing interventions to encourage and promote water intake among children.

## Competing interests

The authors declare that they have no competing interests.

## Authors’ contributions

AD suggested statistical analyses and provided critical input into the manuscript CR conducted the statistical analyses and helped to draft the manuscript FC formulated the research question and reviewed the manuscript. All authors read and approved the final manuscript.

## Acknowledgement

Study supported by Nestle Waters MT.
